# A175 REAL-WORLD EXPERIENCE ON SWITCHING FROM ADALIMUMAB TO BIOSIMILARS IN PATIENTS WITH INFLAMMATORY BOWEL DISEASE – AN OBSERVATIONAL STUDY FROM THREE TERTIARY CARE CENTRES

**DOI:** 10.1093/jcag/gwac036.175

**Published:** 2023-03-07

**Authors:** H Bedi, D Rosenfeld, T Hoang, M Reise-Filteau, B Bressler, Y Leung, S Singh, G Rosenfeld

**Affiliations:** 1 Gastroenterology, University of British Columbia, Vancouver; 2 Western University, London; 3 Medicine, University of British Columbia; 4 Gastroenterology , St. Paul's Hospital, Vancouver; 5 Gastroenterology, University of British Columbia; 6 Gastroenterology, Kelowna General Hospital , Kelowna, Canada

## Abstract

**Background:**

Inflammatory Bowel Disease (IBD) is a chronic inflammatory condition of the bowel which includes ulcerative colitis (UC), Crohn’s disease (CD) or unspecific IBD (IBDu). Adalimumab (ADA), a humanized monoclonal IgG antibody against tumour necrosis factor-alpha (TNFa), is an effective treatment for IBD. Humira^TM^ is an anti-TNFa agent that was approved by Health Canada for the treatment of IBD. In May of 2019, the British Columbia government pharmaceutical benefit plan implemented a biosimilar initiative in May 2019, mandating a non-medical switch from Humira^TM^ to one of the biosimilar drugs.

**Purpose:**

We aimed to evaluate the real-world experience on the comparative safety and effectiveness of adalimumab biosimilar therapy after a mandatory, non-medical switch.

**Method:**

We performed a retrospective chart review of all patients with IBD who either remained on Humira^TM^ or were switched to an adalimumab biosimilar agent, Idacio, at three tertiary care centres. Patients’ demographic data, disease status including CRP and fecal calprotectin before and after switch, and hospital visits or admission after switch were collected. Statistical analysis was performed using ANOVA and t-test.

**Result(s):**

Of the 191 patients included in the study, 145 patients underwent the provincial mandated switch from Humira^TM^ to a biosimilar agent, Idacio® , whereas 46 patients remained on Humira^TM^. The median age at IBD diagnosis was 27 years (range 3-76 years), and at biosimilar switch was 43.5 years (15-78 years). Median disease duration prior to biosimilar switch was 13.5 years (range 0-69 years). 55.1% of patients were male, and 12.9% of patients were active smokers. CD was found in 78.9% of patients, and 19.9% of patients had UC. After biosimilar switch, adverse events, such as rash, nausea, or vomiting, were noted in 10 patients in the biosimilar group, and 7 patients required switch back to Humira^TM^. All patients in the Humira^TM^ group stayed on that therapy. One patient stopped the biosimilar agent due to development of a new cancer requiring chemotherapy. None of the patients required IBD-related emergency department visit or hospital admission. Additionally, there was no difference in CRP or fecal calprotectin values measured before and after the biosimilar switch, and when compared to the patients who stayed on Humira^TM^ (p=0.48, and p=0.142, respectively).

**Conclusion(s):**

We conclude that the clinical benefit of Humira^TM^ was sustained after a non-medical switch to an adalimumab biosimilar. There was no risk of relapse, emergency visit, or hospital admission seen in this study. This is the first Canadian study to establish the safety and efficacy of switch to non-medical switch to an adalimumab biosimilar agent.

**Please acknowledge all funding agencies by checking the applicable boxes below:**

None

**Disclosure of Interest:**

None Declared

